# Early life predictors of subjective cognitive function in older age: emerging longitudinal associations over 6 decades

**DOI:** 10.1093/geronb/gbag180

**Published:** 2026-08-27

**Authors:** Gideon Neidenmark, Catherine Tuvblad, Jonas Persson

**Affiliations:** School of Behavioural, Social, and Legal Sciences, Örebro University, Örebro, Sweden; School of Behavioural, Social, and Legal Sciences, Örebro University, Örebro, Sweden; School of Behavioural, Social, and Legal Sciences, Örebro University, Örebro, Sweden; Aging Research Center (ARC), Karolinska Institute and Stockholm University, Solna, Sweden

**Keywords:** Individual development and adaptation-project, School anxiety, Life-course, Cognitive aging, Metamemory

## Abstract

**Objectives:**

Self-rated memory impairment is common in older age and has been related to objective cognitive decline and dementia. Recently, there has been growing interest in early-life predictors of late-life cognition, but research has been limited due to the absence of data spanning the entire lifespan. The present study investigated whether psychological health, social well-being, socioeconomic status (SES), and cognitive abilities at age 10 and 13 predicted subjective memory almost 6 decades later.

**Methods:**

Psychological health, social well-being, SES, and cognitive abilities were measured with standardized tests and questionnaires at age 10 and 13. Subjective memory, anxiety, and depressive symptoms were measured with the Prospective and Retrospective Memory Questionnaire (PRMQ) and Hospital Anxiety and Depression Scale (HADS) at age 67 (*n *= 625).

**Results:**

In a longitudinal autoregressive structural equation model (SEM), higher levels of psychological health at age 13 predicted lower levels of subjective memory at age 67. This association was independent of current anxiety and depressive symptoms. The model did not provide sufficient support that social well-being, SES, cognitive ability, and school performance at age 13 predicted subjective memory at age 67. Additional analysis revealed that psychological health at age 13, but not at age 10, and anxiety symptoms at age 67 uniquely predicted subjective memory at age 67.

**Discussion:**

These findings extend prior research by showing that psychological health is a robust predictor of subjective memory. Interventions targeting early-life psychological health may help identify individuals with different prospective cognitive outcomes in later life.

Many healthy adults suffer from memory difficulties as they get older (Jonker et al., 2000; Smith et al., 1996). However, some are relatively unaffected, with memory abilities essentially as good as when they were younger (i.e., “successful aging”; Schulz & Heckhausen, 1996). Others show a pattern classified as “usual aging”; decline in abilities that cannot be classified as either pathological or successful (Schulz & Heckhausen, 1996) and some suffer to the extent that they are diagnosed with dementia. Research is still attempting to understand the determinants of individual variability in level and change of cognitive abilities in older age, and a deeper understanding is crucial for promoting the preservation of memory abilities during aging (Hilal & Brayne, 2022).

Despite the variability in objective memory performance, subjective memory impairments may be a part of life for many. The prevalence rate for subjective memory impairments ranges between 10% and 88%, with the highest rates found among the oldest samples (Si et al., 2020). Memory complaints in mid- to late-life have been suggested as a predictor for future memory decline and dementia (Jonker et al., 2000), particularly among those with higher education who are harder to screen for cognitive impairment (van Oijen et al., 2007). However, previous results on whether subjective memory reflects objective memory is mixed. Associations between subjective and objective memory are generally weak, with correlations varying between −0.29 and 0.41 (Crumley et al., 2014), and some have reported non-significant associations (Burmester et al., 2016). Potentially, decline in memory performance, despite being stable over time, may be too subtle to be noticed and reported (Crumley et al., 2014). Alternatively, the decline in memory performance itself may prevent accurate subjective memory reports (Brailean et al., 2019; G. E. Smith et al., 1996). This may explain findings that subjective memory complaints have limited sensitivity value as a diagnostic method for detecting cognitive impairment (Mitchell, 2008). On the other hand, while objective memory assessments are considered the gold standard for detecting cognitive impairment, they have been criticized for not adequately reflecting experiences from everyday life (Steiner & Frey, 2021). Particularly, some suggest that the relationship between subjective and objective memory functions differently in nonexperimental samples and nonlaboratory settings (Jonker et al., 1997). Subjective memory, as subjective reports of memory functioning, may better capture these everyday experiences. This could potentially explain findings that subjective memory complaints may still have specificity value in diagnosing cognitive impairments (Mitchell, 2008). Specifically, subjective memory complaints may help distinguish healthy adults from those with cognitive impairments to a relatively high degree (between 85% and 95%; Mitchell, 2008). However, for these healthy adults, subjective memory can determine whether individuals use effective strategies to improve objective memory (Lachman & Andreoletti, 2006). This could, in turn, affect both well-being and functioning (Verhaeghen et al., 2000). Thus, understanding individual variability in subjective memory specifically may help improve well-being and, potentially, objective memory functioning in later everyday life for those with non-pathological memory abilities.

While objective memory may be associated with subjective memory, other variables may better predict subjective memory. Current cognitive abilities and psychological health both have been linked to subjective memory. For example, individuals with higher levels of attained education may be better equipped to notice subtle changes in cognitive abilities (Schmand et al., 1997; van Oijen et al., 2007), resulting in a higher correlation between subjective and objective memory (Crumley et al., 2014; Hülür et al., 2014). Similarly, intelligence, as an indicator of general cognitive ability, is positively related to subjective memory (Hülür et al., 2014). Likewise, current psychological health may moderate the relationship between subjective and objective memory such that higher depressive symptoms suppress the relationship (Crumley et al., 2014). Poorer current psychological health has been found to account for 20% of the variance in subjective memory (Smith et al., 1996), and subjective memory complaints may reflect poorer psychological health rather than lower objective memory (Jonker et al., 2000). While current levels are associated with subjective memory, it is unclear how these predictors are longitudinally related to subjective memory.

Recently, there has been a growing interest in the importance of early-life and childhood predictors of late-life memory abilities (Jagust, 2016; Walhovd et al., 2023). For example, Karama et al. (2014) demonstrated that cognitive abilities as early as age 11 predict cortical thickness at age 73, which is suggested to be important for successful aging. Furthermore, lifetime exposures accumulate and impact cognitive functioning in later life. Socioeconomic status (SES), for example, shapes opportunities and experiences across the lifespan (Chan et al., 2021), such that individuals with higher SES are more likely to engage in a healthy lifestyle, thereby mitigating cognitive decline and dementia (Röhr et al., 2022). SES tends to cluster with other factors (Hilal & Brayne, 2022), such as lower education (Röhr et al., 2022) and psychological health (McMaughan et al., 2020). Higher childhood SES may also increase social engagement in later life (Hietanen et al., 2016), which can affect both memory (Krueger et al., 2009) and subjective health (Bennett, 2005). Specifically, a cognitively stimulating social environment across the lifespan can help develop strategies that can be used in the face of memory decline in later life (cognitive reserve; Stern, 2012). As such, the clustering of SES with other risk factors and the accumulation of these risk factors over the lifespan pose further risk of cognitive impairment and brain-related decline (Hilal & Brayne, 2022; Röhr et al., 2022). In turn, cognitive impairment and brain-related decline affect quality of life during aging (Jagust, 2016). The literature also suggests that various predictors, such as cognitive abilities, may remain stable across the lifespan (Deary et al., 2004). This accumulation and stability of risk factors across the lifespan underline the importance of investigating early-life predictors of memory variability. In particular, levels already present in childhood may contribute to this accumulation and stability of the risk factors. Yet most previous research has focused on mid- and late-life predictors of late-life cognitive ability due to a lack of data spanning the earliest phases of life, including childhood and adolescence, into older age. There is a need for greater insight into how longitudinal predictors across the lifespan, such as cognitive abilities, psychological health, SES, and social well-being, influence memory abilities, particularly subjective memory, in later life.

The present study is well equipped to address these gaps by using rich longitudinal data from the Individual Development and Adaptation (IDA) project. The IDA project spans 6 decades, with participants first tested at ages 10 and 13 years, with a subsequent follow-up at age 67. This makes the project inimitable in its ability to explore different predictors in early life and their association with subjective memory in later life. Drawing on previous research (e.g., Bennett, 2005; Hilal & Brayne, 2022; Hülür et al., 2014; Smith et al., 1996; Walhovd et al., 2023), we expected that individuals with lower psychological health in the school context, cognitive abilities, social well-being, and SES in early life (at ages 10 and 13) would also show lower levels of subjective memory at age 67.

## Method

### Participants

This study used data from the IDA project, a population-based cohort in Örebro, Sweden. The cohort consists of individuals in school grade 3 in 1965 at the inception of the project (*n *= 1,027; 510 girls and 517 boys), and in grade 6 at the first follow-up in 1968 (*n *= 1,089; 549 girls and 540 boys). Most participants were 10 years old during the first wave and 13 years old at the second wave of data collection. In 2022, all participants who remained in the project, excluding those who previously had actively opted out, could not be contacted, or were deceased, were invited to participate in a new wave of data collection. Out of these, 625 (349 women and 276 men) participated in the data collection in 2022, when the majority of the participants were 67 years old.

### Measurements

Subjective memory is the outcome in the present study and was measured with the Swedish version of the PRMQ (Rönnlund et al., 2008; Smith et al., 2000). The current study utilized 15 available items on everyday instances of forgetting and includes both prospective and retrospective aspects of subjective memory. An example item is “Do you forget something that you were told a few minutes before?.” All items were rated on a 5-point response scale, with “Never” coded as 1 and “Very often” as 5. To create scale scores, the mean score on the individual items was calculated for each participant, with the lowest possible score being 1 and the highest being 5. No distinction was made between subscales for this study, partly due to the high correlation between the subscales (*r *= 0.90). Reliability was *ɷ* = 0.89.

Psychological health in the school context was measured with three items from the student questionnaires administered at ages 10 and 13, reflecting school anxiety. An example question is “Do you have difficulty keeping up with the pace in school?” (for the complete set of items see Supplementary Materials). For the questionnaire administered at age 10 years, all items were scored on a 4-point scale and were recoded, with 1 coded as never and 4 as always. For the questionnaire administered at age 13 years, all items were scored on a 5-point scale, with 1 coded as “Almost never” and 5 as “Often”/“Almost”/“Always.” Reliability analysis found *ɷ* = 0.52 for age 10 and *ɷ* = 0.56 for age 13.

Social well-being was measured using four items from the student questionnaires administered to participants aged 10 and 13. An example question is “Do you like your peers in your class?.” For the questionnaire administered in 1965, the response scale varied across the items (between 3 and 4-point scales), with 3–4 coded as “Yes”/“Always”/“All” and 1 as “No”/“Never”/“None.” For the questionnaire administered in 1968, all items were administered on a 5-point scale with 5 coded as “Always”/“Almost always”/“All of them,” and 1 as “Rarely”/“Almost never”/“Almost none.” Items were re-coded so that a higher score reflected lower social well-being, and a lower score reflected higher social well-being. Reliability analysis found *ɷ* = 0.60 for age 10, and *ɷ* = 0.64 for age 13.

Socioeconomic status was measured through a combination of items administered either to the participants or their parents in 1965 and 1968. These items included information regarding whether the participant had their own room, what type of house they lived in, the educational level of the family, and the family income (e.g., “Do you have your own room or space at home where you can be alone and have your things?”). The response options were recoded for each item to reflect either a higher (1) or lower (0) SES. Items were categorized as higher if the family income per month >20.000 SEK, parents had attained a degree higher than high school, participants had their own space/room, and the family resided in a house or a townhouse. Items were categorized as lower if the family income per month ≤20.000 SEK, if the parents had attained education below or equivalent to a high school degree, if the participants did not have their own space/room, and if the family resided in a rented home. The scores for each of these items were summed up (highest score = 4; lowest score = 0). As such, a higher score reflected higher SES, and a lower score reflected lower SES.

Cognitive ability was measured with the differential cognitive assessment ([begåvningsanalys; DBA]; for more information see Bergman et al., 2014; Magnusson & Backteman, 1978). The DBA test consisted of 6 subscales across three cognitive domains: verbal ability (similarities, opposites), inductive reasoning (letter combinations, figure series), and spatial ability (blocks, and steel folding). The number of correct responses for each subscale reflected the score of the participant on that specific subscale. The reliability has previously been found to be *α* = 0.93 (see Bergman et al., 2014).

School performance was measured with standardized tests of two school subjects: Swedish and mathematics. These tests were used to measure scholastic achievement in a Swedish national perspective and contained different aspects of the curriculum. The highest possible scores in grade 3 were 89 for Swedish and 52 for mathematics. The highest possible scores in grade 6 were 103 for Swedish and 70 for mathematics.

School performance was also measured by grades in Swedish, mathematics, and religion (i.e., Christianity) at ages 10 and 13. These grades were obtained through official school records. The lowest possible grade in each subject was 1, and the highest grade was 5.

Current anxiety and depressive symptoms were measured with the Swedish version of the Hospital Anxiety and Depression Scale (HADS; Lisspers et al., 1997; Zigmond & Snaith, 1983) in 2022 when participants were 67 years old. This scale consists of 14 questions about anxiety and depressive symptoms. The items were rated on a 4-point response scale, with “Not at all” coded as 0 and “Most of the time” as 3. To create scale scores, the mean score on the individual items for each subscale was calculated for each participant, with 0 as the lowest possible score and 3 as the highest on both subscales. Reliability was *ɷ* = 0.84 and *ɷ* = 0.73 for the Anxiety and Depressive subscales, respectively. The correlation between the subscales was *r *= 0.54.

Subjective health was measured using a single item (“How would you describe your own health?”) in 2022 when participants were 67 years old. The item was rated on a 5-point scale, with “Very bad” coded as 1 and “Very good” as 5.

### Procedure

The data utilized in the present study were collected across three separate waves in 1965, 1968, and 2022. Data from 1965 and 1968 were collected in school settings in Örebro, Sweden. Participants underwent extensive testing, including standardized tests on school subjects, intelligence tests, and questionnaires. In addition, the participants’ parents completed questionnaires in 1965 and 1968. Due to the context of the testing, students who were absent or not part of the Örebro school district at the first wave were added to the cohort in the second wave.

For the 2022 data collection, a questionnaire was sent out to all participants in the IDA project. The questionnaire included various measures, including PRMQ, HADS, and subjective health. The invitation to participate was sent by mail and prompted participants to complete the questionnaire either online or in paper format (*n *= 235 and 390, respectively). As several reminders were mailed out over a period of 1 year, a few participants accidentally filled out more than one copy (particularly one online and one in paper format). When this occurred, the online response was selected.

### Ethics

At the inception of the IDA-project, it operated with the approval and funding of the Swedish School Board, and participants took part as part of their education. At that time, written informed consent was not required from the children/adolescents or their parents under the legal framework. Participation was voluntary, and individuals could withdraw from the project at any time and for any reason. The ethics of the IDA project were reviewed by the Humanities and Social Sciences Research Council in 1986, which granted a positive evaluation. Since then, participants have provided informed consent for continued participation during subsequent data collections. The 2022 data collection was approved by the Swedish Ethical Review Authority (dnr. 2022-02563-01, dnr. 2022-06123-02, and dnr. 2025-00802-02), and written consent was obtained from all participants.

### Statistical methods

All data analysis was performed using R 4.4.3 (R Core Team, 2025) in RStudio (RStudio Team, 2026) with the Lavaan package (Rosseel et al., 2025). Unstandardized composite scores were used for reporting variable descriptives and dropout analyses. For the structural equation models (SEM), latent variables were created to account for measurement error for school anxiety, social well-being, school grades, cognitive ability, and subjective memory. For cognitive ability, standardized DBA domain composites of the subscale total scores were added as indicators reflecting three separate components of cognitive ability (namely, inductive, spatial, and verbal ability). As described earlier, SES was measured with individual items consisting of parents’ and participants’ reports; therefore, it was entered as a composite observed variable. Similarly, standardized tests of school performance and subjective health were entered as a composite observed variable. Inference was based on nonparametric bootstrapped standard errors and percentile confidence intervals computed with 20.000 resamples for robust uncertainty estimates.

Assumptions were tested beforehand, and there were violations of multivariate normality. However, univariate normality showed only mild deviations, with a few indicators showing skewness. Additionally, some outliers were found but not removed from the analyses because they were not severely influential. Measurement invariance was tested for the relevant latent variables, and metric invariance was supported for school anxiety, social well-being, and school grades. Full Information Maximum Likelihood was used to handle missing data, and Maximum Likelihood was used for estimating the SEM models.

## Results

Descriptive statistics of unstandardized composites are presented in Table 1. Correlations between latent constructs and observed composites are found in Table 2. Attrition analyses were conducted for gender/sex and each predictor at ages 10 and 13 between those who participated in the most recent data collection and those who dropped out between the initial and the most recent data collections (Supplementary Table 1). These analyses revealed that the participants who dropped out had significantly lower psychological health (at age 10 and 13), social well-being (at age 13), lower SES (at age 10), lower grades (at age 10 and 13), lower scores on standardized tests of school performance (at age 10 and 13), and were more likely to be male compared to those who remained in the study.

**Table 1:** Descriptives for predictors and outcome.

Variable	*N*	Mean	*SD*	Min	Max	Skew	Kurtosis
PRMQ	620	1.84	0.48	1.00	4.20	0.77	1.26
School anxiety age 10	447	1.61	0.48	1.00	3.33	0.67	0.22
School anxiety age 13	470	1.50	0.59	1.00	4.33	1.40	1.99
Social well-being age 10	447	1.59	0.50	1.00	3.33	0.97	0.70
Social well-being age 13	470	1.88	0.57	1.00	4.00	0.85	1.19
SES age 10	625	1.66	1.02	0.00	4.00	0.51	−0.45
SES age 13	625	1.92	1.05	0.00	4.00	0.37	−0.75
Cognitive ability age 10^a^	476	−0.01	0.81	−3.03	2.20	−0.36	0.71
Cognitive ability age 13^a^	491	0.00	0.81	−2.84	1.50	−0.64	0.14
School grades age 10	471	3.29	0.77	1.33	5.00	−0.12	−0.47
School grades age 13	494	3.33	0.86	0.00	5.00	−0.40	0.41
Standardized tests of school performance age 10	468	38.62	11.10	0.00	64.00	−0.74	1.64
Standardized tests of school performance age 13	495	47.28	20.14	0.00	85.00	−0.96	0.49
HADS anxiety	623	0.49	0.46	0.00	2.57	1.12	1.14
HADS depression	623	0.32	0.30	0.00	1.71	1.40	2.23
Subjective health	616	3.67	0.86	1.00	5.00	−0.42	0.12

*Note*. HADS = hospital anxiety and depression scale; PRMQ = prospective and retrospective memory questionnaire; SES = socioeconomic status. Based on unstandardized composite scores.

a Composite of standardized subscale total scores.

**Table 2:** Correlation matrix of predictors and outcome.

Variable	1	2	3	4	5	6	7	8	9	10	11	12	13	14	15	16	17
1 PRMQ																	
2 School anxiety age 10	0.19*																
3 School anxiety age 13	0.34***	0.39**															
4 Social 5 well-being age 10	0.13	0.19*	0.09														
5 Social well-being age 13	0.03	0.17	0.22**	0.27*													
6 SES age 10^a^	0.01	−0.05	−0.09	0.02	−0.06												
7 SES age 13^a^	−0.01	−0.01	−0.13*	0.08	−0.05	0.50***											
8 Cognitive ability age 10	<0.01	−0.16*	−0.22**	−0.01	−0.08	0.26***	0.24***										
9 Cognitive ability age 13	0.01	−0.13	−0.20**	0.03	−0.14	0.29***	0.26***	0.89***									
10 School grades age 10	−0.04	−0.27***	−0.30***	−0.06	−0.13	0.17***	0.14**	0.88***	0.94***								
11 School grades age 13	−0.02	−0.16**	−0.28***	<0.01	−0.11	0.20***	0.19***	0.80***	0.88***	0.87***							
12 Standardized tests of school performance age 10^a^	−0.02	−0.17**	−0.22***	0.07	−0.07	0.18***	0.16***	0.74***	0.79***	0.82***	0.71***						
13 Standardized tests of school performance age 13^a^	−0.04	−0.14*	−0.22***	0.03	−0.07	0.14***	0.15***	0.63***	0.66***	0.58***	0.61***	0.48***					
14 HADS anxiety	0.44***	0.08	0.09	0.08	−0.02	−0.04	−0.08*	−0.02	−0.04	−0.01	−0.03	−0.03	−0.06				
15 HADS depression	0.38***	0.06	0.12*	0.08	0.09	−0.06	−0.10**	−0.03	−0.07	−0.05	−0.09	< −0.01	−0.07	0.54***			
16 Subjective health	−0.28***	−0.18**	−0.17**	−0.01	−0.10	0.01	0.06	0.06	0.07	0.10	0.13**	0.10*	0.06	−0.28***	−0.41***		
17 Gender/sex	0.03	0.13*	0.03	−0.03	−0.01	0.19***	0.06	0.04	0.14**	0.10*	0.10*	0.02	0.05	0.09*	−0.06	0.02	

*Note*. HADS = hospital anxiety and depression scale; PRMQ = prospective and retrospective memory questionnaire; SEM = structural equation model; SES = socioeconomic status. Correlations are fully standardized estimates from a covariance SEM; latent constructs as entered in the SEM.

a Standardized composite.

* *p *≤ .05.

** *p *≤ .01.

*** *p *≤ .001.

### Main analysis

To investigate whether early-life psychological health in the school context, social well-being, SES, and cognitive ability predict subjective memory in later life, a longitudinal autoregressive latent SEM was used. School anxiety, social well-being, SES, cognitive ability, school grades, and standardized tests of school performance were added as predictors of subjective memory. Each included construct was measured at two time points (at ages 10 and 13) using the same indicators. Metric invariance was imposed across time for school anxiety, social well-being, and school grades. All constructs from age 13 were regressed on their corresponding construct at age 10 to test autoregressive stability over time. The constructs from age 13 were then regressed on PRMQ measured at age 67, thereby testing prospective associations while modeling temporal carryover. The constructs from ages 10 and 13, respectively, were correlated within time points, and residuals of indicators were correlated across time. Gender/sex and subjective health ratings at age 67 were included as covariates.

Results for the model are presented in Table 3 and Figure 1. Model fit was CFI = 0.88, TLI = 0.87, RMSEA = 0.04, SRMR = 0.07. The model explained 18% variance in PRMQ at age 67 (*R*^2^ = 0.18). There were autoregressive stability found between age 10 and 13 for school anxiety (*B *= 0.55, 95% CI [0.26, 0.85], *β* = 0.43), social well-being (*B *= 0.37, 95% CI [0.14, 0.60], *β* = 0.30), SES (*B *= 0.50, 95% CI [0.42, 0.57], *β* = 0.50), cognitive ability (*B *= 0.79, 95% CI [0.69, 0.87], *β* = 0.94), school grades (*B *= 0.93, 95% CI [0.83, 1.02], *β* = 0.85), and standardized tests of school performance (*B *= 0.48, 95% CI [0.36, 0.60], *β* = 0.49), indicating substantial carryover across waves. There was not sufficient support that gender/sex predicted PRMQ at age 67 (*B *= 0.03, 95% CI [−0.06, 0.12], *β* = 0.05), as the confidence interval included zero and the estimated effects were close to zero. However, we did find sufficient support that subjective health ratings at age 67 predicted PRMQ at age 67 (*B *= −0.16, 95% CI [−0.22, −0.11], *β* = −0.30). Results also showed that school anxiety at age 13 prospectively predicted PRMQ at age 67 (*B *= 0.35, 95% CI [0.20, 0.56], *β* = 0.34). This indicates that lower levels of psychological health in the school context were associated with lower subsequent levels of subjective memory. We did not find sufficient support that social well-being (*B *= −0.04, 95% CI [−0.16, 0.10], *β* = −0.04), SES (*B *= 0.02, 95% CI [−0.03, 0.07], *β *= 0.03), cognitive ability (*B *< 0.01, 95% CI [−0.05, 0.03], *β* = 0.03), school grades (*B *= 0.07, 95% CI [−0.09, 0.29], *β* = 0.11), or standardized tests of school performance (*B *= −0.01, 95% CI [−0.08, 0.06], *β* = −0.01) prospectively predicted PRMQ, as their confidence intervals included zero and effects were estimated close to zero. Thus, only early-life psychological health prospectively predicts subjective memory in later life. We did not find support that early-life social well-being, SES, cognitive ability, or school performance prospectively predict subjective memory in later life.

**Figure 1:**
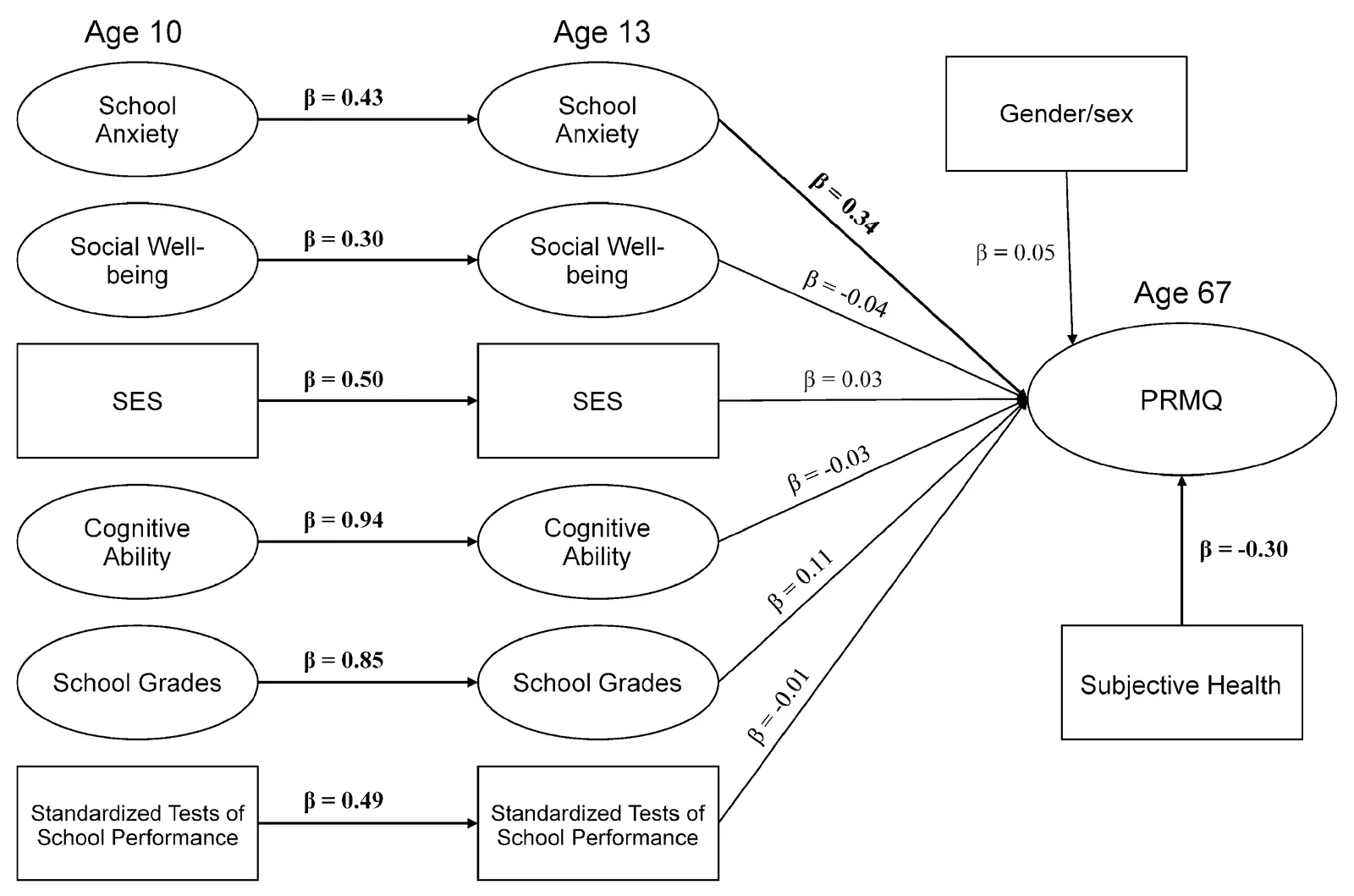
Path diagram of longitudinal autoregressive SEM. Latent variables are represented by circles and observed variables by rectangles. All predictors within the same time point are correlated. Estimates for autoregressive and predictive paths were included. Residual correlations between corresponding indicators across time were omitted for clarity. Full measurement model can be made available upon request. PRMQ = prospective and restrospective memory questionnaire; SEM = structural equation model; SES = socioeconomic status.

**Table 3:** Results of the longitudinal autoregressive SEMs.

Predictor	Autoregressive paths				Predictive effects on PRMQ			
	***β***	*B*	*SE*	95% CI (bootstrap, *B*)	***β***	*B*	*SE*	95% CI (bootstrap, *B*)
School anxiety	0.43	0.55	0.15	[0.26, 0.85]	0.34	0.35	0.10	[0.20, 0.56]
Social	0.30	0.37	0.12	[0.14, 0.60]	−0.04	−0.04	0.07	[−0.16, 0.10]
SES	0.50	0.50	0.04	[0.42, 0.57]	0.03	0.02	0.03	[−0.03, 0.07]
Cognitive ability	0.94	0.79	0.05	[0.69, 0.87]	−0.03	< −0.01	0.02	[−0.05, 0.03]
School grades	0.85	0.93	0.05	[0.83, 1.02]	0.11	0.07	0.11	[−0.08, 0.29]
Standardized tests of school performance	0.49	0.48	0.06	[0.36, 0.60]	−0.01	−0.01	0.03	[−0.08, 0.06]
Gender/sex					0.05	0.03	0.05	[−0.06, 0.12]
Subjective health					−0.30	−0.16	0.03	[−0.22, −0.11]

*Note*. CFI = comparative fit index; CI = confidence interval; PRMQ = prospective and retrospective memory questionnaire; RMSEA = root mean square error of approximation; SEMs = structural equation models; SES = socioeconomic status; SRMR = standardized root mean square residual; TLI = Tucker-Lewis index. Standardized and unstandardized estimates of predictors of latent PRMQ based on 19,994 successful bootstrap runs. *R*^2^ = 0.18 and model fit indices: CFI = 0.88, TLI = 0.87, RMSEA = 0.04, SRMR = 0.07.

### Exploratory sensitivity analyses with current anxiety and depressive symptoms

As previous literature has established that current psychological health is robustly associated with subjective memory, we performed sensitivity analyses with current anxiety and depressive symptoms to establish whether the effects persist. For this purpose, two separate autoregressive SEM were constructed. Each model was set up identically to the primary model described previously, with the addition of either current anxiety or depressive symptoms. To maintain model parsimony and reduce complexity, anxiety and depressive symptoms were added as separate composites in separate models.

Results from both sensitivity models were consistent with the main model such that the magnitude and direction of the estimated effects remained largely unchanged after adjusting for current anxiety and depressive symptoms (see Supplementary Materials). Adding anxiety symptoms at age 67 increased the proportion of explained variance (*R*^2^ = 0.26–0.31). Additionally, we found that anxiety (*B *= 0.46, 95% CI [0.33, 0.59], *β* = 0.86) and depressive symptoms at age 67 (*B *= 0.57, 95% CI [0.40, 0.75], *β* = 0.33) were associated with PRMQ at age 67. Therefore, adjusting for current anxiety and depressive symptoms did not alter the conclusions from the main model.

### Exploratory analysis of the relative influence of psychological health across lifespan

Given that psychological health in early-life and current anxiety and depressive symptoms were found to predict subjective memory, we also explored the unique predictive effects of the timing of psychological health on subjective memory. For this purpose, a longitudinal latent SEM was utilized with school anxiety at age 10 and 13, and anxiety symptoms at age 67 as predictors of PRMQ at age 67. Predictors were allowed to covary, and metric invariance was imposed on school anxiety at age 10 and 13. Residuals of indicators for school anxiety were correlated across time.

Results of the longitudinal latent SEM can be found in Table 4. Model fit was CFI = 0.91, TLI = 0.90, RMSEA = 0.05, SRMR = 0.04. The model explained 32% of the variance in PRMQ at age 67 (*R*^2^ = 0.32). Results showed that school anxiety at age 13 (*B *= 0.31, 95% CI [0.17, 0.48], *β* = 0.28) and anxiety symptoms at age 67 (*B *= 0.55, 95% CI [0.40, 0.70], *β* = 0.45) uniquely predicted PRMQ at age 67. This indicates that lower levels of psychological health in the school context at age 13 and more current anxiety symptoms were associated with lower levels of subjective memory at age 67, even after controlling for school anxiety at age 10. We did not find sufficient support that school anxiety at age 10 (*B *= 0.05, 95% CI [−0.14, 0.25], *β* = 0.04) predicted PRMQ beyond school anxiety at age 13 and anxiety symptoms at age 67, as the confidence interval included zero. This suggests that both psychological health in early-life and current anxiety symptoms uniquely predict subjective memory in later life, beyond the shared influence of psychological health. Furthermore, this indicates that while early-life psychological health predicts subjective memory beyond current anxiety symptoms, prepubescent psychological health may not predict subjective memory beyond this.

**Table 4:** Results of the exploratory longitudinal latent SEMs.

Predictor	***β***	*B*	*SE*	95% CI (bootstrap, *B*)
School anxiety age 10	0.04	0.05	0.10	[−0.14, 0.25]
School anxiety age 13	0.28	0.31	0.08	[0.17, 0.48]
HADS anxiety subscale age 67	0.45	0.55	0.08	[0.40, 0.70]

*Note*. CFI = comparative fit index; CI = confidence interval; HADS = Hospital anxiety and depression scale; RMSEA = root mean square error of approximation; SEMs = structural equation models; SRMR = standardized root mean square residual; TLI = Tucker-Lewis index. Standardized and unstandardized estimates of Timing based on 19,999 successful bootstrap runs. *R*^2^ = 0.32 and model fit indices: CFI = 0.91, TLI = 0.90, RMSEA = 0.05, SRMR = 0.04.

## Discussion

The present study examined early-life predictors of subjective memory in later life. Out of the six included predictors, we found evidence that psychological health in the school context in early life (ages 10 and 13) predicted subjective memory at age 67. Additional exploratory analyses demonstrated that this relationship persists for psychological health at age 13, even after controlling for current anxiety and depressive symptoms. This finding extends previous research (Derouesné et al., 1999; Hülür et al., 2015; Smith et al., 1996) by demonstrating that psychological health in early-life acts as a robust predictor of subjective memory later in life.

Previous research has shown that higher levels of depressive symptoms (Brailean et al., 2019), anxiety (Derouesné et al., 1999; Steinberg et al., 2013), and stress (Steinberg et al., 2013) are all related to lower subjective memory. Previous studies that have examined the relationship between objective and subjective memory have reported mixed findings, and this inconsistency could possibly be attributed to a high incidence of depressive symptoms in individuals with lower subjective memory (Jonker et al., 2000). For example, Derouesné et al. (1999) found lower subjective memory in younger compared to older adults, but this could likely be explained by lower levels of reported psychological health in younger individuals. The present results further expand this previous knowledge by showing that lower levels of psychological health already present in early-life can act as a predictor of later-life subjective memory. In particular, pubescent psychological health may influence later-life subjective memory. Additionally, we found support that this association remained even after controlling for current anxiety and depressive symptoms. Therefore, our results suggest that psychological health in early life uniquely contributes to later development, beyond current psychological health. This is supported by previous findings that childhood, and particularly adolescence, is a critical period for development which influences aging outcomes (Hilal & Brayne, 2022; Jagust, 2016; Walhovd et al., 2023). As such, our results expand previous knowledge in demonstrating that psychological health in early life may uniquely influence later-life memory abilities.

Previous research has shown that psychological health may remain stable across the lifespan. For example, the accumulation of early-life stressors (Lähdepuro et al., 2019), neuroticism (Gale et al., 2011) and anxious/withdrawal behavior (Monk et al., 2023) are associated with higher symptoms or risk of anxiety disorders in later life, which may not attenuate with age (Clark et al., 2010). Likewise, Colman et al. (2007) found that about 80% of their sample had stable trajectories of anxiety symptoms, from no symptoms to continuous severe symptoms over the lifespan. This together would indicate that psychological health, particularly anxiety symptoms, remains fairly stable over the lifespan. Though this may be the case, our analyses show that early-life psychological health is still predictive of subjective memory in later life, beyond influences from current anxiety and depressive symptoms. That is, early-life psychological health uniquely influences subjective memory beyond common effects from psychological health present across the lifespan. However, it is worth noting that psychological health and anxiety and depressive symptoms were not operationalized in the same way, and the findings might therefore be influenced by measurement differences.

Interestingly, although social well-being (Krueger et al., 2009), SES (Chan et al., 2021), and cognitive abilities (Deary et al., 2022) are often considered predictors of objective memory, we found no evidence that they predict subjective memory. However, given the relatively low reliability of some of our measures, it is possible that the apparent lack of effects is due to measurement problems rather than a lack of effects. Nonetheless, it is also possible that this reflects the weak correlation between subjective and objective memory that is often reported (Crumley et al., 2014). It could be argued that objective and subjective memory reflect different constructs or tap into different parts of memory. Alternatively, it could reflect measurement differences between subjective and objective memory. In particular, subjective memory is known to be subject to bias while objective memory is subject to ecological validity problems (Crumley et al., 2014). For example, lower psychological health has been associated with more negative evaluations and enhanced self-monitoring of one’s abilities (Brailean et al., 2019). This suggests that subjective memory may not accurately reflect objective memory. In fact, previous research suggests that subjective memory reflects psychological health rather than objective memory (Jonker et al., 2000). However, previous research has shown that subjective memory complaints can act as a predictor and indicator of future diagnosis of dementia in the absence of decline on objective tests (Jessen et al., 2014). This may especially be the case for highly educated individuals who are harder to screen for cognitive decline (van Oijen et al., 2007).

The study has several strengths, including a relatively large sample, inclusion of both cognitive and non-cognitive predictors of subjective memory, and availability of longitudinal data spanning almost 6 decades. However, some limitations should be acknowledged. First, our measurements had relatively low reliability, which also varied across the time points, and between different predictors. Therefore, it is also possible that the measures did not adequately measure the intended constructs, especially for school anxiety, social well-being, and SES, which were based on only a few questions each. For example, Krueger et al. (2009) reported that different components of social well-being can have implications for objective memory. It is, therefore, possible that the lack of sufficient support for the predictors here may be due to measurement properties. Second, we did not consider any interaction or multimodal effects between predictors. Some research suggests that this is an important area of future research for understanding subjective memory (Vaillant & Mukamal, 2001). Third, our study is also limited in that it does not include longitudinal measurements of subjective memory or estimates of the predictors in later life. This has implications for making causal claims about the relationships of the measures included. It is possible that subjective memory remains stable over the lifespan, or that subjective memory influences psychological health. For example, higher ratings of subjective memory have been associated with a lower risk of developing depressive symptoms (Ji et al., 2023). While our study cannot establish a causal relationship between subjective memory and psychological health, our results may point to psychological health as a potential influence on subjective memory rather than the other way around. Since the outcome measure was based on survey data, measures of current objective memory and dementia screening were not available. We can therefore not conclude whether psychological health predicts subjective memory beyond current cognitive ability. Finally, we found systematic differences in attrition rates. Similar to most longitudinal studies, selection bias is likely present in the current study. Therefore, it is important to be cautious in drawing conclusions regarding the generalizability of the results. Likewise, it is possible that the estimated associations are affected by selection bias. For example, it might exaggerate the relationship between psychological health and subjective memory. Regardless, future research should consider longitudinal measurements of both subjective memory and its potential predictors, such as mid-life mediators of these relationships.

In the present study, we demonstrated that psychological health in childhood and adolescence predicted subjective memory in older age, up to 54–57 years later. Based on these results, it would be interesting to explore whether psychological health in early life is causally linked to subjective memory in later life. There may in fact be a reciprocal relationship between psychological health and subjective memory (Schmand et al., 1997), and it is unclear whether poor psychological health in early life leads to lower subjective memory. Future research with designs that would support causal conclusions is needed. Furthermore, previous research suggests that risk factors for dementia and brain-related decline cluster together (Hilal & Brayne, 2022), creating inequalities during aging. As such, we recommend that future research explore whether predictors similar to those examined in this study interact and cluster over the lifespan, and whether this in turn influences subjective memory.

More generally, our findings, together with future studies, could potentially contribute to new knowledge on when, how, and what we should target in interventions for preserving or even improving self-reported memory ability in older age. Previous research has struggled to find effective interventions to improve subjective memory, whether that be exercise (Zuniga et al., 2016) or focused memory training (Frankenmolen et al., 2018). Our findings highlight the importance of psychological health, particularly during early life in the school context, for self-reported memory ability in later life. Interventions that target childhood psychological health could be beneficial not only for subjective memory but also have positive effects outside of the cognitive domain.

## Supplementary Material

gbag180_Supplementary_Data

## Data Availability

Data and analytic code can be provided for replication purposes upon request. The study was not preregistered.
