# Supplementary material for: Early life predictors of subjective cognitive function in older age: emerging longitudinal associations over 6 decades
Source: J Gerontol B Psychol Sci Soc Sci. 2026 Aug 27;81(11):gbag180. doi: 10.1093/geronb/gbag180 (PMC13653334; doi:10.1093/geronb/gbag180)
Supplement: Supplementary file 1 [file gbag180_supplementary_data.docx]

***The Journals of Gerontology, Series B: Psychological Sciences and Social Sciences* Supplementary Material: Neidenmark, Tuvblad, & Persson. Early life predictors of subjective cognitive function in older age: Emerging longitudinal associations over six decades.**

**Contents**

[Supplementary Table 1. T-tests, Chi-square Tests, and Descriptive Statistics for Comparing Drop-out Sample and Sample	2](#_Toc237539121)

[Supplementary Table 2. Results of the Sensitivity Analyses with Anxiety and Depressive Symptoms from Age 67	3](#_Toc237539122)

[Supplementary Table 3. Items Administered to Participants in 1965 and 1968 Reflecting School Anxiety	4](#_Toc237539123)

[Supplementary Table 4. Items Administered to Participants in 1965 and 1968 Reflecting Social Well-being	5](#_Toc237539124)

# Supplementary Table 1. T-tests, Chi-square Tests, and Descriptive Statistics for Comparing Drop-out Sample and Sample

| **Variable** | **Longitudinal Drop-out** | | |
| --- | --- | --- | --- |
|  | ***N*** | **Mean Dropout** | **Test Statistic** |
| School Anxiety Age 10 | 518 | 1.67 | -2.02* |
| School Anxiety Age 13 | 567 | 1.60 | -2.74** |
| Social Well-being Age 10 | 518 | 1.62 | -0.83 |
| Social Well-being Age 13 | 567 | 1.96 | -1.98* |
| SES Age 10 | 767 | 1.53 | 2.23* |
| SES Age 13 | 767 | 1.83 | 1.70 |
| Cognitive Ability Age 10 ^a^ | 549 | -0.01 | 0.18 |
| Cognitive Ability Age 13 ^a^ | 598 | 0.00 | 0.00 |
| School Grades Age 10 | 529 | 2.99 | 6.12*** |
| School Grades Age 13 | 607 | 2.91 | 7.65*** |
| Standardized Tests of School Performance Age 10 | 518 | 34.38 | 5.85*** |
| Standardized Tests of School Performance Age 13 | 607 | 40.50 | 5.51*** |
| Gender/sex | 550 |  | 19.60*** |

*Note*. Based on unstandardized composite scores. Longitudinal Dropout refers to participants who did not participate in the most recent data collection used for the outcome variable (*n* = 767).

^a^ Composite of standardized subscale total scores.

**p* ≤ 0.05, ***p* ≤ 0.01, ****p* ≤ 0.001.

# Supplementary Table 2. Results of the Sensitivity Analyses with Anxiety and Depressive Symptoms from Age 67

|  |  | **Autoregressive Paths** | | | |  | **Predictive Effects on PRMQ** | | | |
| --- | --- | --- | --- | --- | --- | --- | --- | --- | --- | --- |
|  | **Predictor** | **β** | **B** | **SE** | **95% CI (bootstrap, B)** |  | **β** | **B** | **SE** | **95% CI (bootstrap, B)** |
| **Original Model** | School Anxiety | 0.43 | 0.55 | 0.15 | [0.26, 0.85] |  | 0.34 | 0.35 | 0.10 | [0.20, 0.56] |
|  | Social | 0.30 | 0.37 | 0.12 | [0.14, 0.60] |  | -0.04 | -0.04 | 0.07 | [-0.16, 0.10] |
|  | SES | 0.50 | 0.50 | 0.04 | [0.42, 0.57] |  | 0.03 | 0.02 | 0.03 | [-0.03, 0.07] |
|  | Cognitive Ability | 0.94 | 0.79 | 0.05 | [0.69, 0.87] |  | -0.03 | <-0.01 | 0.02 | [-0.05, 0.03] |
|  | School Grades | 0.85 | 0.93 | 0.05 | [0.83, 1.02] |  | 0.11 | 0.07 | 0.11 | [-0.08, 0.29] |
|  | Standardized tests of School Performance | 0.49 | 0.48 | 0.06 | [0.36, 0.60] |  | -0.01 | -0.01 | 0.03 | [-0.08, 0.06] |
|  | Gender/sex |  |  |  |  |  | 0.05 | 0.03 | 0.05 | [-0.06, 0.12] |
|  | Subjective Health |  |  |  |  |  | -0.30 | -0.16 | 0.03 | [-0.22, -0.11] |
| **Adjusted for Anxiety Symptoms** | School Anxiety | 0.42 | 0.54 | 0.15 | [0.24, 0.83] |  | 0.32 | 0.33 | 0.09 | [0.18, 0.53] |
|  | Social | 0.30 | 0.37 | 0.12 | [0.15, 0.60] |  | -0.02 | -0.02 | 0.06 | [-0.13, 0.11] |
|  | SES | 0.50 | 0.50 | 0.04 | [0.42, 0.56] |  | 0.05 | 0.03 | 0.02 | [-0.02, 0.07] |
|  | Cognitive Ability | 0.94 | 0.79 | 0.05 | [0.69, 0.87] |  | <-0.01 | 0.00 | 0.02 | [-0.04, 0.03] |
|  | School Grades | 0.85 | 0.93 | 0.05 | [0.83, 1.01] |  | 0.06 | 0.04 | 0.09 | [-0.11, 0.21] |
|  | Standardized tests of School Performance | 0.49 | 0.48 | 0.06 | [0.36, 0.60] |  | 0.01 | 0.01 | 0.03 | [-0.06, 0.07] |
|  | Gender/sex |  |  |  |  |  | -0.01 | -0.01 | 0.04 | [-0.09, 0.07] |
|  | Subjective Health |  |  |  |  |  | -0.15 | -0.09 | 0.03 | [-0.15, -0.04] |
|  | Anxiety Symptoms |  |  |  |  |  | 0.40 | 0.46 | 0.07 | [0.33, 0.59] |
| **Adjusted for Depressive Symptoms** | School Anxiety | 0.43 | 0.55 | 0.15 | [0.25, 0.85] |  | 0.34 | 0.34 | 0.10 | [0.20, 0.56] |
|  | Social | 0.30 | 0.37 | 0.12 | [0.14, 0.60] |  | -0.06 | -0.06 | 0.06 | [-0.18, 0.07] |
|  | SES | 0.50 | 0.50 | 0.04 | [0.42, 0.56] |  | 0.05 | 0.03 | 0.02 | [-0.02, 0.08] |
|  | Cognitive Ability | 0.94 | 0.79 | 0.05 | [0.69, 0.87] |  | -0.05 | -0.01 | 0.02 | [-0.05, 0.03] |
|  | School Grades | 0.85 | 0.93 | 0.05 | [0.83, 1.01] |  | 0.12 | 0.08 | 0.10 | [-0.06, 0.30] |
|  | Standardized tests of School Performance | 0.49 | 0.48 | 0.06 | [0.36, 0.60] |  | 0.00 | 0.00 | 0.03 | [-0.07, 0.07] |
|  | Gender/sex |  |  |  |  |  | 0.04 | 0.04 | 0.04 | [-0.04, 0.13] |
|  | Subjective Health |  |  |  |  |  | -0.13 | -0.08 | 0.03 | [-0.14, -0.03] |
|  | Depressive Symptoms |  |  |  |  |  | 0.33 | 0.57 | 0.09 | [0.40, 0.75] |

*Note*. Standardized and unstandardized estimates of predictors of latent PRMQ for original model are based on 19994 successful bootstrap runs, for model adjusted for anxiety symptoms are based on 19999 successful bootstrap runs, and for model adjusted for depressive symptoms are based on 19998 successful bootstrap runs. For original model: *R^2^* = 0.18 and model fit indices: CFI = 0.88, TLI = 0.87, RMSEA = 0.04, SRMR = 0.07 for original model. For model adjusted for anxiety symptoms: *R^2^* = 0.31, CFI = 0.88, TLI = 0.87, RMSEA = 0.04, SRMR = 0.07. For model adjusted for depressive symptoms: *R^2^* = 0.26, CFI = 0.88, TLI = 0.87, RMSEA = 0.04, SRMR = 0.07.

# Supplementary Table 3. Items Administered to Participants in 1965 and 1968 Reflecting School Anxiety

|  | **School Anxiety Age 10** | | |  | **School Anxiety Age 13** | | |
| --- | --- | --- | --- | --- | --- | --- | --- |
| **Items** | **Swedish** | **English Translation** | **Response Scale** |  | **Swedish** | **English Translation** | **Response Scale** |
| 1 | Händer det att Du har ont i magen på morgonen innan Du går till skolan? | Do You ever have a sore tummy in the mornings before going to school? | Never (1)  Often (2)  Sometimes (3)  Always (4) |  | Händer det att Du har ont i magen på morgonen innan Du går till skolan? | Do You ever have a sore tummy in the mornings before going to school? | Almost never (1)  Occasionally (2)  Quite often (3)  Often (4)  Almost always (5) |
| 2 | Händer det att du mår illa på morgonen innan Du går till skolan? | Do You ever feel sick in the morning before going to school? | Never (1)  Often (2)  Sometimes (3)  Always (4) |  | Händer det att Du mår illa på morgonen innan Du går till skolan? | Do You ever feel sick in the morning before going to school? | Almost never (1)  Not so often (2)  Sometimes (3)  Rather often (4)  Often (5) |
| 3 | Har Du svårt att hinna med att göra allt vad fröken säger? | Do You find it difficult to have time to do everything the teacher tells You to do? | Never (1)  Often (2)  Occasionally (3)  Always (4) |  | Har Du svårt att hinna med arbetstakten i skolan? | Do You have difficulty keeping up with the pace in school? | Almost never (1)  Rarely (2)  Occasionally (3)  Rather often (4)  Often (5) |
| Omega | 0.52 | | |  | 0.56 | | |

# Supplementary Table 4. Items Administered to Participants in 1965 and 1968 Reflecting Social Well-being

|  | **Social Well-being Age 10** | | |  | **Social Well-being Age 13** | | |
| --- | --- | --- | --- | --- | --- | --- | --- |
| **Items** | **Swedish** | **English Translation** | **Response Scale** |  | **Swedish** | **English Translation** | **Response Scale** |
| 1 | Trivs Du med Dina kamrater i klassen? | Do You like your classmates? | Yes (1)  Sometimes (2)  No (3) |  | Trivs Du bra med Dina kamrater i klassen? | Do You get along well with your peers in the class? | Always (1)  Most times (2)  Sometimes (3)  Not particularly well (4)  Rarely (5) |
| 2 | Har Du roligt på rasterna? | Do You have fun at break time? | Always (1)  Often (2)  Sometimes (3)  Never (4) |  | Har Du trevligt på rasterna? | Do You enjoy breaktime? | Almost always (1)  Often (2)  Occasionally (3)  Rarely (4)  Almost never (5) |
| 3 | Hur många av Dina kamrater trivs Du med i klassen? | How many of Your classmates do You feel comfortable with? | All (1)  The majority (2)  A few (3)  None (4) |  | Hur många av Dina kamrater trivs Du med i klassen? | How many of Your classmates do you like? | All of them (1)  Most of them (2)  Some of them (3)  One or two (4)  Almost none (5) |
| Omega | 0.60 | | |  | 0.64 | | |
